# Evaluation of Artificial Dermis for the Treatment of Leg Ulcers: Clinical Outcomes From an Exploratory Study

**DOI:** 10.1111/iwj.70739

**Published:** 2025-08-06

**Authors:** Vincent Casoli, Laura De Luca, Emilie Desnouveaux, Efterpi Demiri, Fabiana Battaglia, Francesco Stagno d’Alcontres, Gabriele Delia

**Affiliations:** ^1^ Service de Chirurgie Plastique Reconstructrice et esthétique, CFX MICHELET CHU de Bordeaux, Université de Bordeaux Bordeaux France; ^2^ Papageorgiou Hospital (Department of Plastic Surgery) Aristotle University of Thessaloniki Thessaloniki Greece; ^3^ Department of Plastic Surgery Università degli Studi di Messina, AOU Policlinico G. Martino Messina Italy

**Keywords:** chronic leg ulcer, dermal regeneration, health‐related quality of life, IDRT, skin graft, wound healing

## Abstract

Chronic leg ulcers present a significant clinical challenge due to their prolonged healing time and high recurrence rates. This prospective, multi‐centre, non‐randomised, observational study investigated the efficacy of a dermal regeneration template in improving skin graft integration for chronic leg ulcer treatment. Thirty patients were enrolled, with a control group receiving only skin grafts to evaluate the additional benefits of the template. Patients were assessed for pain levels, healing rates, wound retraction, pruritus, dressing type, analgesic use, complications, surgeon‐evaluated wound recovery using the Vancouver scale, quality of life through the EuroQol questionnaire and photographic wound documentation. At 18 months, 70.0% of patients achieved at least a 50% reduction in wound surface area and 56.7% experienced complete wound closure. Significant improvements were observed in pain and discomfort (*p* = 0.0125), mobility (*p* = 0.0267), pain levels (*p* = 0.0340), vascularity (*p* = 0.0275) and overall wound reduction (*p* = 0.0368). The control group demonstrated lower wound reduction and complete healing rates, reinforcing the superior effectiveness of the dermal regeneration template in combination with skin grafting. This study highlights the potential of this approach to accelerate wound healing, reduce patient discomfort and enhance quality of life compared to traditional skin grafting alone.

**Summary**

- Chronic leg ulcers are difficult to heal and frequently recur, creating a substantial clinical and economic burden.
- This multicenter observational study evaluated the use of Integra Dermal Regeneration Template (IDRT) combined with split‐thickness skin grafting in 30 patients.
- At 18 months, 70% of patients achieved ≥ 50% wound area reduction, and 56.7% experienced complete wound closure.
- Significant improvements were observed in pain, mobility, vascularity, and overall wound healing compared to skin grafting alone.
- IDRT combined with skin grafting may enhance dermal regeneration, improve graft take, and provide a safe and effective option for managing complex chronic leg ulcers.

## 1 Introduction

The skin acts as a critical barrier against thermal loss, dehydration and microbial invasion. Therefore, prompt and effective wound healing is essential to maintaining physiological stability and optimising clinical outcomes [1].

According to literature, CLU prevalence in the general population ranges from 0.045% to 0.63%, reaching 3.6% in people older than 65 years [2].

Chronic venous leg ulcers affect between 500 000 and 2 million persons annually in the United States and account for more than 50% of leg ulcers [3, 4].

Early venous interventions have been shown to improve healing and reduce recurrence, as demonstrated by the ESCHAR and EVRA trials [5, 6]. However, not all patients are eligible for such procedures due to anatomical or clinical constraints.

Their prevalence is predicted to increase as the population ages and risk factors, such as obesity and congestive heart failure, become more prevalent [7]. The public health burden and rapidly increasing costs of chronic ulcer care, including their complications, reflect a lack of an effective therapy for these disabling lesions [8].

Chronic wounds are soft tissue defects that do not heal through predictable stages and they are typically characterised by a healing time greater than 4 weeks [9]. Their treatment often requires both extensive reconstructive operations and prolonged use of expensive dressings, resulting in protracted hospitalisations, high infection rates and graft loss. The recent emergence of acellular dermal matrices represents a new reconstructive option for the management of chronic wounds [10].

The integra dermal regeneration template (IDRT) used in this study is a bilayer matrix consisting of cross‐linked bovine type I collagen and chondroitin‐6‐suphate, covered by a semi‐permeable silicone layer. This structure is designed to mimic the extracellular matrix, promote fibroblast migration and angiogenesis and support neodermis formation. The matrix has been used in clinical settings for over a decade and is distinct from synthetic scaffolds in its biological origin and regenerative mechanism [11]. This artificial skin replacement system promotes fibroblast migration and leads to the creation of a new dermis [12, 13, 14, 15]. Several studies focusing on chronic ulcer treatment with dermal substitutes have been reported [16, 17, 18, 19]. Nevertheless, there is scant literature on evaluating short‐ and long‐term outcomes of wound healing time and quality of healed tissue in multi‐centre prospective studies.

The aim of this study was to determine whether the use of IDRT would improve skin graft take rate in the surgical treatment of CLUs.

The evaluated hypothesis was that IDRT leads to the formation of a highly vascularised dermis that facilitates skin graft take.

The principal objective of the study was to evaluate the efficacy of IDRT in the treatment of CLUs. In this study, the use of IDRT would be considered effective if the outcome was wound reduction ≥ 50% during the 18 months following the dermo‐epidermal grafting.

Secondary endpoints included: (1) proportion of patients with complete healing at 18 months (complete healing was defined as reduction > 90% of the wound surface, a threshold commonly used in chronic wound studies to account for minimal residual defects that typically re‐epithelialize spontaneously); (2) pain evaluation at Day 15, month 3, 6, 12 and 18; (3) proportion of patients with no recurrence at the end of the follow‐up (18 months); (4) oxygen pressure increase in the tissues surrounding the ulcer; (5) complications; (6) functional outcomes and (7) patient's QoL evaluation.

Patient‐specific details such as Ankle‐Brachial Pressure Index (ABPI), type and dosage of medication (e.g., vascular or immunosuppressive drugs) and relevant comorbidities such as diabetes and hypertension were documented. These factors were integrated into the analysis to ensure accurate characterisation of the study population.

## 2 Patients and Methods

### 2.1 Study Design and Patients

This was a prospective, multi‐centre, non‐randomised, observational Phase IV study, based on the IDRT indications (CE mark) and on HAS guidelines for the treatment of CLUs (NCT01285973).

No formal sample size calculation was performed, as the study was designed to explore clinical outcomes in a real‐world setting. The number of patients enrolled was based on feasibility and the aim to provide preliminary multi‐centre evidence on the use of IDRT in chronic leg ulcers.

Ethical approval was obtained from each participating institution's Institutional Review Board (IRB), ensuring compliance with the principles outlined in the 1975 Declaration of Helsinki, as revised in 2013.

Before participation, all patients provided written informed consent and the study adhered to the guidelines for human experimentation. The protocol followed the International Organisation for Standardisation (ISO) 14 155:2011 for clinical investigations of medical devices in human subjects.

The study was approved under protocol RECON‐EMEA‐10 (NCT01285973). The Clinical Study Report was finalised on 9 May 2017, after data analysis had been completed.

A total of 30 patients were enrolled from three different centres (France, Italy and Greece), according to the following inclusion criteria: patients of either sex, age between 18 and 90 years, presence of a lower leg ulcer, ulcer duration greater than 6 months or ulcer area > 10 cm^2^, surgeon's recommendation for IDRT implantation, ineligibility for skin flap surgery, affiliated to or beneficiary of the social security system and signed informed consent to participate in the observational study.

The diagnosis of venous ulcer was established through clinical criteria and confirmed by duplex ultrasound in all patients.

Patient selection and treatment planning were performed by multi‐disciplinary teams including plastic and vascular surgeons at each centre.

Prior to enrolment, all subjects had undergone standard venous treatments, including compression therapy and, when indicated, correction of superficial venous reflux. Patients were considered eligible only if ulcers persisted despite these interventions and were no longer amenable to additional venous procedures. Compression therapy was not systematically applied during the study period due to local contraindications or patient intolerance. All patients underwent a comprehensive venous disease assessment prior to inclusion in the study. The diagnosis of venous ulcer was confirmed by duplex ultrasonography in each case, in accordance with international guidelines for the classification and treatment of chronic venous insufficiency. The ultrasound evaluation included examination of superficial, deep and perforating veins to assess the presence and extent of venous reflux. Patients with significant arterial disease or mixed aetiology ulcers were excluded from the study population.

Among the 30 patients enrolled, 5 received only skin grafting without prior IDRT implantation and constituted the control group. The remaining 25 patients underwent treatment with both IDRT and split‐thickness skin graft.

Patients included in the observational study were those with chronic leg ulcers that, according to the clinical recommendations of the Haute Autorité de Santé (HAS, France), required a skin graft rather than standard dressings or compression therapy. All treatments were administered in accordance with routine clinical practice.

Treatment decisions, including whether to use IDRT or standard skin grafting, were made entirely by the clinicians at each centre based on individual patient characteristics. The study did not assign or influence treatment allocation in any way.

The study did not influence treatment decisions, which were made by the clinicians based on patient‐specific clinical indications.

These recommendations suggest grafting in ulcers unresponsive to conventional care, especially when conservative management is precluded by exposure of tendons or bony structures, or when there is insufficient granulation tissue to support graft take [20].

Furthermore, previous unsuccessful attempts with conventional skin grafts were documented at baseline in several patients. This information helped assess patient eligibility for IDRT, particularly in light of previous failed interventions.

The main exclusion criteria were the presence of a circumferential ulcer, active wound infection, immunosuppression, confirmed allergy to bovine collagen, glycosaminoglycans and/or silicone, pregnancy, patients under legal guardianship, patients whose health conditions would impair follow‐up for at least 18 months or whose mental health would compromise completion of the self‐evaluation questionnaires or the wound located in an area not visible to the patient (as no self‐assessment would be possible).

In the study, only one wound per patient was studied. When a patient presented with two ulcers eligible for surgery, a random allocation procedure was used to choose which ulcer would be included.

A control group of patients was also included, where only skin grafts were applied without prior IDRT implantation. Patients in this group were carefully monitored for any adverse effects and assessed for conditions such as allergies to collagen, glycosaminoglycans or silicone, as well as any pre‐existing conditions that might hinder treatment or increase the risk of complications. Additionally, their medication intake was closely monitored to ensure compatibility with the treatment protocol.

This control group served as a direct comparator, allowing the evaluation of the additional benefits provided by IDRT in comparison to traditional skin grafting.

Group assignment was not randomised. Patients were assigned to the control group when IDRT was not available at the treating centre or when they did not meet all the conditions required for IDRT application, such as local availability, timing or surgeon decision based on ulcer characteristics.

This study was carried out in a real‐world clinical conditions. Group assignment and sample size were determined pragmatically based on treatment availability, patient characteristics and feasibility across participating centres.

A post hoc power analysis was performed for the primary endpoint, defined as the proportion of patients achieving a ≥ 50% wound surface reduction during follow‐up. This threshold was selected based on prior studies identifying a ≥ 50% reduction as a clinically meaningful improvement in chronic wound healing. Based on the observed response rate of 70% (21 out of 30 patients) and using a one‐sided *z* test against a clinically relevant threshold of 50%, the estimated statistical power was 48.0% (*α* = 0.05).

Although the power is below the conventional threshold of 80%, this study was designed as a multi‐centre, observational Phase IV investigation to generate preliminary data in real‐world clinical settings. The large effect size observed nonetheless supports the clinical relevance of the findings and provides a rationale for future confirmatory studies with adequate sample size.

### 2.2 Baseline Visit

After signing the informed consent form, subjects underwent the baseline visit with a clinical examination and collection of the following information: concomitant disease(s), treatments, past medical and surgical history, allergies, wound history, pain assessment (self‐administered visual analogue scale), wound location, wound and exudate description (qualitative and quantitative), wound edges, surrounding skin and wound assessment in terms of colour, fibrin, necrotic tissue and granulation.

**FIGURE 1:**
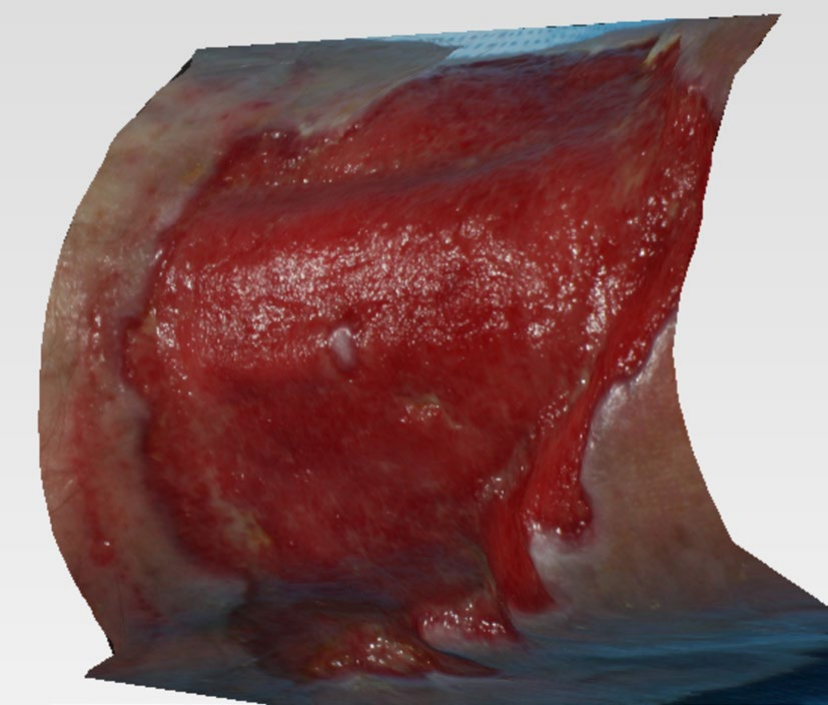
Use of QuantifiCare 3D reconstruction to measure size of the wounds.

Additional assessments included measurement of transcutaneous oxygen pressure (TcPO_2_) and/or Laser Doppler, serum chemistry profile, wound culture, wound photographic documentation (Quantificare instrument, measurement of length, width, depth and surface area with a 3D reconstruction software) (Figure 1).^1^

An EuroQoL questionnaire was used to evaluate the quality of life perceived by patients. This questionnaire is composed of a scale categorised into 3 levels: level 1 indicating no problems and level 2 and 3 indicating increasing issues.

### 2.3 First Procedure: IDRT Implantation

The following information was collected: wound measurement (length, width and depth) and photography (Quantificare instrument) and type of dressing and complications. The IDRT was shaped with scissors and fixed to the wound edges with staples. Negative pressure wound therapy (NPWT) was applied in 11 out of 30 patients (36.7%) following IDRT placement. The choice between NPWT and standard dressing (e.g., vaseline gauze) was based on ulcer depth, exudate amount and surgeon preference. NPWT was favoured in cases with high exudate or when the matrix required more secure fixation. Reapplication of IDRT was required in three patients (10%) due to partial matrix detachment or suboptimal integration, as judged clinically. These decisions were made intraoperatively or within the first 2 weeks, based on wound appearance and lack of matrix adherence.

### 2.4 Post‐IDRT Follow‐Up Visits

The initial dressing was changed on the first to fifth post‐operative day. Following this, dressings were replaced one to two times per week. Pain assessment (VAS), the subject's general state, changes to the wound or dressings (complication, dermis colour and type of dressing) and photographic documentation were performed at each visit.

### 2.5 Second Procedure: Dermo‐Epidermal Graft

Patients underwent a dermo‐epidermal graft once the IDRT matrix had been incorporated, signalled by a peach colour throughout the matrix and a loosening of the silicone sheet. The silicone sheet was removed and a skin graft (0.19 mm thick in average), harvested with a dermatome from the medial aspect of the thigh, was placed over the neodermis and fixed with staples.

Then, the following assessments were performed: proportion of IDRT graft take, neodermal appearance, area and thickness of the skin graft, type of dressing applied, complications and wound photography.

### 2.6 Post‐Grafting Follow‐Up Visits

The initial post‐grafting dressing was replaced on the first to fifth post‐operative day. Thereafter, dressings were replaced one to two times per week. Pain assessment (VAS), patient's general state, recipient site healing (visual estimate of healing percentage, skin retraction, pruritus), type of dressing, analgesic treatment, complications and wound photography were performed at each visit.

### 2.7 Mid and Long‐Term Follow‐Up Visits

A follow up visit was performed at 3, 6, 12 and 18 months post‐graft. The following assessments were done: pain assessment (VAS), subject's general state, ulcer healing (visual estimate of healing percentage, skin retraction, pruritus), type of dressing used (if any), analgesic treatment, complications, quality of ulcer healing as evaluated by the surgeon (Vancouver Scar Scale [VSS]), QoL questionnaire and functional discomfort evaluated by the patient (EuroQoL) and wound photography.

Measurements of TcPO_2_ and/or Laser Doppler were done at Months 6, 12 and 18. Global functional and aesthetic results (surgeon's and patient's opinions) were evaluated at Month 18.

Device‐related adverse events (DRAEs) were observed in five patients, all of whom were treated with IDRT (5/25; 20%). A total of six DRAEs were recorded, consisting mainly of localised infections at the graft or matrix site. All cases were managed locally and did not result in systemic infection or graft failure.

No adverse events were reported in the control group (0/5).

Additionally, four patients died during the follow‐up period, all from the IDRT group. These deaths were unrelated to the medical device and were attributed to severe pre‐existing conditions, including congestive heart failure and chronic pulmonary disease. Wound healing was evaluated using a combined approach: objective analysis with a 3D stereophotogrammetry system (Quantificare, France) and visual clinical assessment. 3D imaging was used at baseline and immediately prior to grafting to obtain precise measurements of the wound surface area and depth. Follow‐up wound assessment was performed by visual estimation due to logistical limitations in accessing the 3D imaging system at all visits. To ensure consistency, each centre assigned the same trained clinician to perform follow‐up evaluations. These clinicians were trained in wound assessment protocols before study initiation and standard criteria were used to classify healing stages and surface reduction. Inter‐observer variability was minimised by using photographic documentation and standardised visual scoring sheets.

The follow‐up duration of 18 months was chosen to allow for a comprehensive evaluation of not only primary wound healing but also long‐term outcomes such as tissue quality, recurrence and functional results. While most studies focus on endpoints within 12–24 weeks, chronic leg ulcers often exhibit delayed healing and high recurrence rates. A longer follow‐up period enabled the detection of late complications, sustained closure and patient‐reported outcomes. However, we acknowledge that prolonged follow‐up may introduce confounding variables and this limitation is discussed accordingly.

## 3 Results

### 3.1 Study Subjects

**FIGURE 2:**
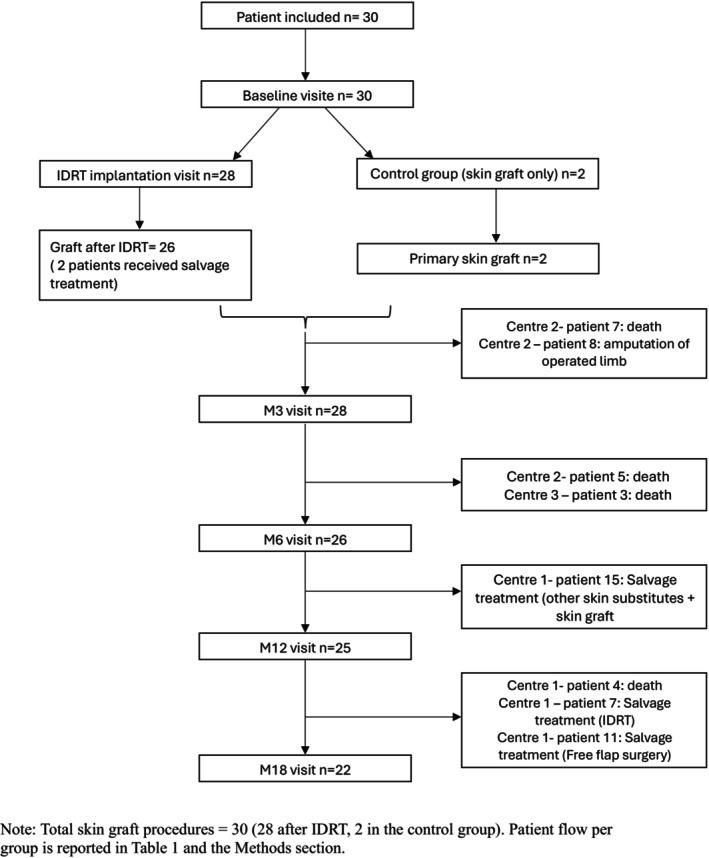
CONSORT statement. *Note:* Total skin graft procedures = 28 (23 after IDRT, 5 in the control group). Patient flow per group is reported in Table 1 and the Section 2.

Between April 2022 and December 2023, 30 patients were enrolled. Of these, 28 patients received the IDRT and a split‐thickness skin graft. The overall early discontinuation rate was 26% (*n* = 8; four deaths, one amputation and three salvage treatments) (Figure 2).

An in‐depth analysis ruled out any direct link between the deaths and the surgical procedures or the use of IDRT. The deaths were attributed to significant pre‐existing comorbidities such as heart failure and chronic pulmonary disease.

The demographic characteristics of enrolled subjects are listed in Table 1.

**TABLE 1:** Patient demographics.

	*N* = 30 (16 male)			
	Age (years)	Height (cm)	BMI (kg/m^2^)	Weight (kg)
Mean	60.8	167.1	25.6	71.8
SD	19.0	10.4	5.1	17.5
Median	64.0	167.5	25.1	71.0
Min; max	24.0; 86.0	145.0; 185.0	14.1; 35.2	37.0; 108.0

Regarding the concomitant disorders, 23.3% of the population had diabetes, 46.8% had high blood pressure, one subject (3.3%) had hepatitis C, 46.7% had a vascular pathology, including chronic venous insufficiency, post‐thrombotic syndrome and compensated peripheral arterial disease and 23.3% had an immune pathology. Nine patients (30%) were smokers. The mean VAS pain rating (100 mm scale) at baseline was 29.9; 9 patients (30%) had no pain, 18 (60%) had intermittent pain and 3 (10%) had constant pain.

The comparison between patients who received only skin grafts and those treated with IDRT and skin grafts showed that the control group had significantly lower rates of wound healing and wound area reduction.

Baseline characteristics of the control group were as follows: median age 63 years (range 42–85), three males and two females. Comorbidities included diabetes (one patient), hypertension (two patients) and vascular pathology (two patients). Median ulcer size was 68 cm^2^ (range 32–110 cm^2^), located in the lower third of the leg in all cases. Median ulcer duration was 4.5 months. No patients in the control group had received prior treatment with IDRT.

Only 20% of the control group achieved more than 50% wound reduction by 18 months and none achieved complete healing, confirming the superior efficacy of the IDRT combination therapy.

### 3.2 Wounds Characteristics

The wound aetiology was venous.

The median wound duration was 5.9 months (range 0.4–356.4 months).

The most frequent location was the lower leg (83.3%), particularly the lower third in 60% and right leg in 56.7%.

Mean ulcer area was 74.6 cm^2^ and 0.6 cm deep.

Wound cultures were obtained at baseline in 73.3% of the patients, on average 19.8 days before the baseline visit and there was bacterial growth in 77.3% of wounds.

Pre‐operative NPWT was applied in 33.3% of the wounds for a mean duration of 15.5 days; 53.3% of ulcers had had prior surgical preparation and 10 subjects had received antibiotic treatment prior to the Baseline visit.

### 3.3 IDRT and Skin Graft Procedures

IDRT was fixed with staples (93.3%) and either NPWT (36.7%) or vaseline dressing ( ± antiseptic) was applied.

**FIGURE 3:**
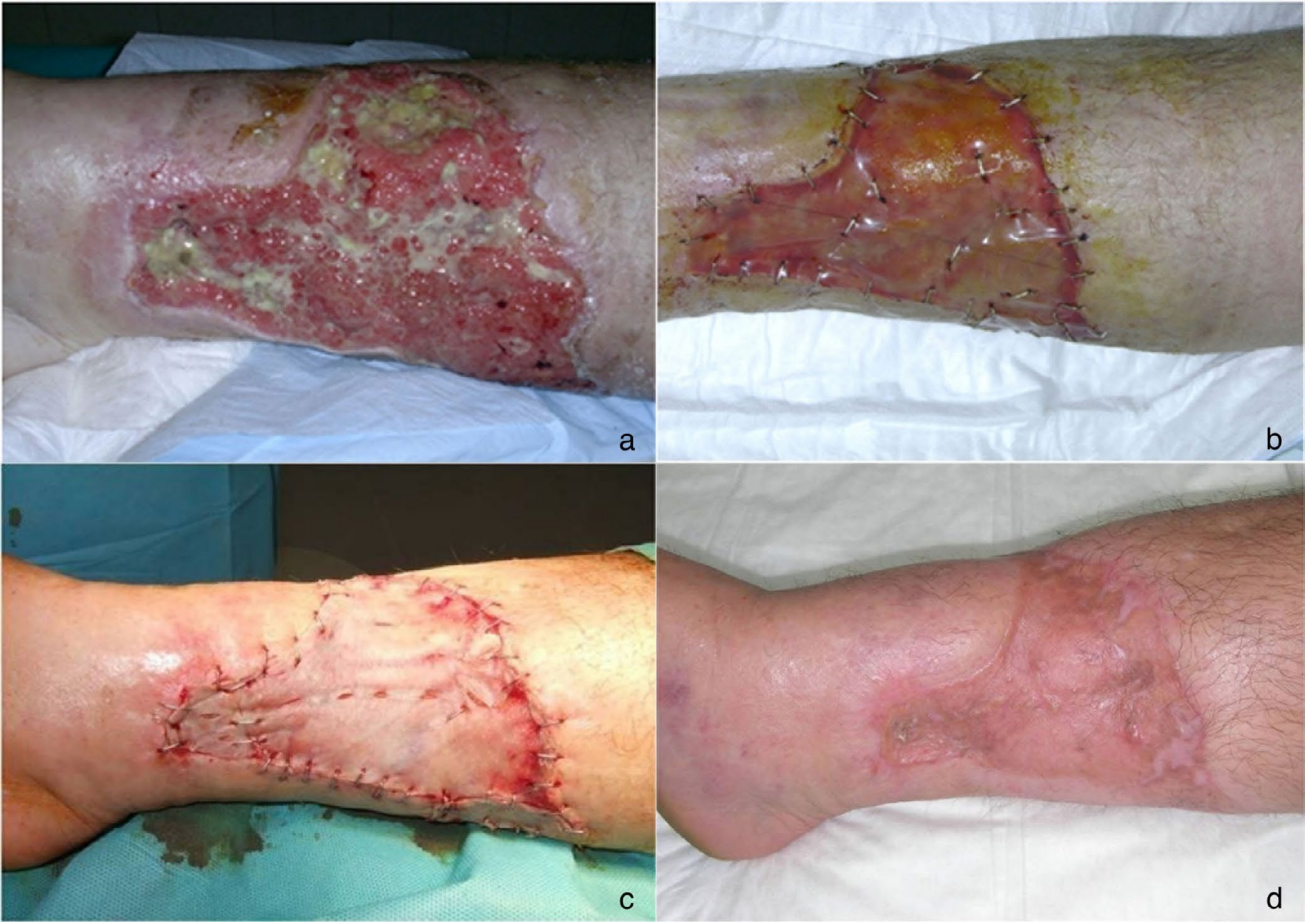
Diabetic ulcer with wide infection (a), surgical toilet and application of VAC therapy, positioning of dermal substitute (b), dermo‐epidermic graft (c), 1‐year results (d).

**FIGURE 4:**
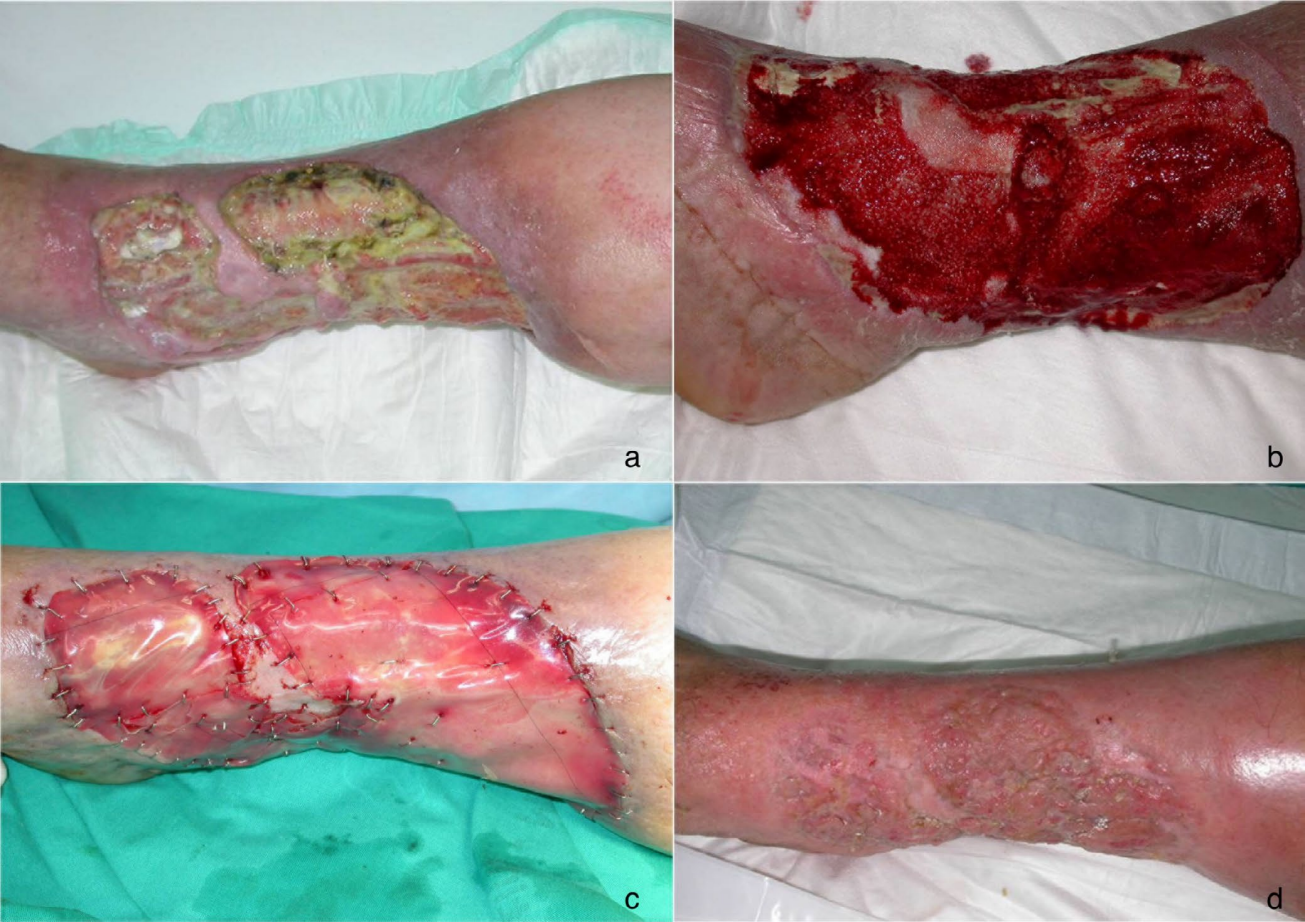
Venous ulcera in diabetic patient (a), surgical toilet and application of vac therapy (b), positioning of dermal substitute (c), dermo‐epidermic graft, 6‐month result (d).

**FIGURE 5:**
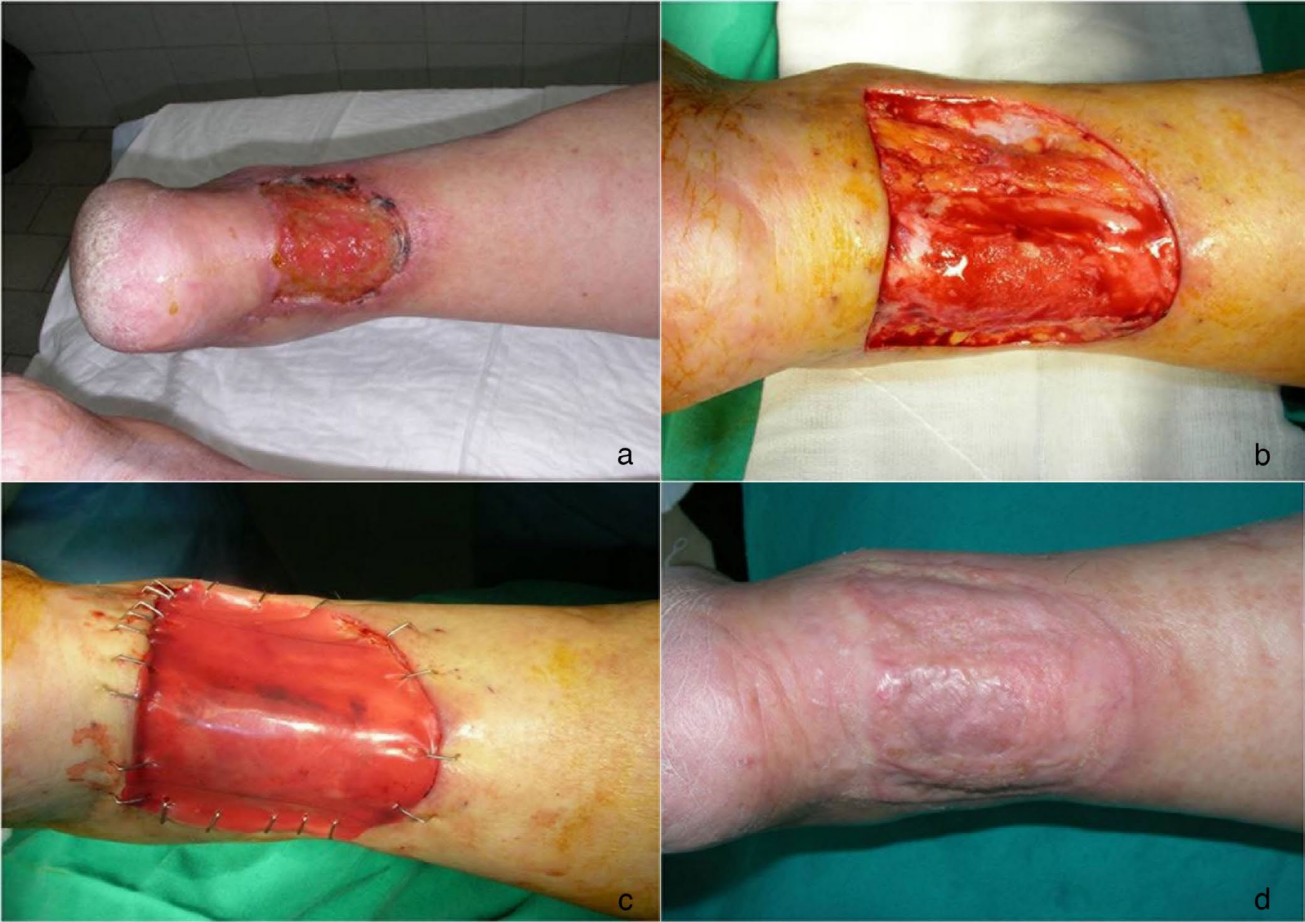
Microangiopathic ulcer (a), surgical toilet and application of vac therapy (b), positioning of dermal substitute (c), dermo‐epidermic graft, 6‐month result (d).

On the day of skin grafting (31 ± 7 days after placement), 77.7% of the ulcers had an IDRT take rate greater than 70% (4 ulcers had less than 70% and 23 had 70% or more). The neodermis was completely revascularised in 89% of wounds. The neodermis was lightly abraded with a curette and brush in 66.7% of the wounds, at the discretion of the investigator. The thigh was the donor site in 89% of the patients and skin graft thickness was about 0.19 mm, on average. Skin graft was fixed mostly with staples (89%) (Figures 3, 4, 5).

Spontaneous reepithelialisation occurred in one patient (3.3%), who did not require split‐thickness skin grafting. This case was documented and confirmed by clinical and photographic assessment.

### 3.4 Effectiveness

Seventy percent of subjects (*n* = 21) achieved > 50% reduction in wound area by 18 months (Table 2).

**TABLE 2:** Primary effectiveness criteria (missing‐failure imputation).

	Total (*N* = 30)
Greater than 50% reduction in the surface area of the wound	21 (70.0%)
95% confidence interval	[50.6%; 85.3%]
*p*‐value of comparison to reference practice, 70%	*p* = 0.5685
*p*‐value of comparison to 50% (proportion beneath which the new technique would not be deemed to be useful)	*p* = 0.0214

*Note:* No measurement was possible in four subjects due to the quality of the photographs.

No measurements were available in four cases and five subjects did not achieve this rate.

Regarding the secondary endpoints, during the study, 56.7% of subjects had complete healing (wound closure > 90%) (Table 3), with only three subjects (10%) having a re‐opening wound.

**TABLE 3:** Proportion of subjects with complete wound healing.

	*N* = 30
Complete healing (reduction during follow‐up > 90%) during the study and follow‐up	17 (56.7%)
95% confidence interval	[38.9%; 74.4%]
Complete healing (reduction > 90%) at M18	12 (40.0%)
95% confidence interval	[22.5%; 57.5%]

At 18 months, 40% of subjects had complete healing (four subjects died and two had no proper photography).

Concerning wound surface, as measured with the 3D photographic software, the mean reduction rate increased over the follow‐up period with 54.4%, 75.9%, 83.7% and 91.9% reduction at 3, 6, 12 and 18 months, respectively.

Mean healing rate, as visually assessed by the investigator during the follow‐up visits was 73.8%, 73.3%, 88.5% and 89.9% at 3, 6, 12 and 18 months, respectively.

Skin retraction was observed in 30% of the wounds. More than half of the subjects reported dry and sensitive skin between 6 and 18 months follow‐up and only 5% of subjects reported pruritus at 18 months.

VSS was only completed after the wound was healed. Fourteen subjects had VSS assessed at Months 3 and 19 had VSS assessed at Month 18. The mean VSS decreased from 4.21 at Month 3 to 3.58 at Month 18.

Initial VAS pain score was 29.9 and progressively decreased over time (19.74, 13.18, 13.26 and 11.80 at 3, 6, 12 and 18 months, respectively), although the intake of analgesics remained stable across the study with 37.5%, 40.9%, 50% and 38.1% of subjects receiving analgesics at 3, 6, 12 and 18 months, respectively. Subjects experienced decreasing levels of discomfort as the follow‐up progressed. Overall, only 20%–28% of subjects experienced significant or major discomfort.

TcPO_2_ and laser Doppler measurement showed no significant changes between treated and contralateral limb.

At the 18‐month follow‐up, most of the surgeons and subjects were satisfied or very satisfied with the functional (76.2% of surgeons and subjects) and aesthetic (71.4% subjects and 66.6% surgeons) results.

The EuroQoL scale is evaluated. In this study, as follow‐up progressed, there was an improvement in each of the tool's five categories (mobility, self‐care, usual activities, pain and discomfort, anxiety and depression) with an increased proportion of subjects indicating a Level 1 or 2 and a substantial decrease of Level 3.

At 18 months, no Level 3 ratings were reported for mobility, self‐care and anxiety/depression. We observed a significant time effect for EuroQoL pain and discomfort (*p* = 0.0125), EuroQoL mobility (*p* = 0.0267), pain‐VAS (*p* = 0.0340), vancouver vascularity (*p* = 0.0275) and wound reduction *(p* = 0.0368).

## 4 Discussion

In this study, we showed that 70.0% of the subjects achieved at least a 50% reduction in wound area. Moreover, 60.7% of subjects, with a mean wound duration of 38.8 months, reached complete wound healing within 18 months. Treatment of complex chronic wounds combining the IDRT and skin‐graft showed a strong speed up of the healing process, leading to a wound surface reduction rate of 54.5% at 3 months after surgery.

A comparison of these results with the Bordeaux team's historic experience (i.e., 70%), in terms of wound area reduction was made and there was no significant difference between them (*p* = 0.5685). Although the proportion of patients with > 50% wound area reduction was similar to Bordeaux's historic cohort, differences in patient complexity and ulcer characteristics may account for outcome variability. Additional benefits observed with IDRT, including improved tissue quality and patient‐reported outcomes, suggest a broader clinical impact beyond surface healing alone.

A comparison between patients treated with only skin grafts (control group) and those treated with IDRT and skin grafts was made. In the control group, the healing rate and wound area reductionwere assessed.

Although skin grafting is not routinely indicated in standard venous ulcers due to high recurrence and limited benefit, all patients in our cohort presented with chronic ulcers unresponsive to prior conservative management. In many cases, the presence of exposed anatomical structures (e.g., tendons, bone) precluded continued compression therapy or spontaneous healing. Thus, skin grafting was employed as part of a reconstructive approach in complex, treatment‐resistant ulcers, in line with certain salvage protocols.

Only 20% of the control group achieved more than 50% reduction in wound area by 18 months, and none achieved complete healing. This highlights the superior efficacy of the combined IDRT and skin graft approach over the skin graft alone.

Clinically, we considered that if IDRT resulted in < 50% closure, it would not be useful in our practice. However, IDRT outcomes were significantly better (*p* = 0.0214) than this apriori threshold.

This threshold was chosen based on the, particularly, difficult‐to‐treat nature of the study population. All patients had chronic leg ulcers refractory to standard treatments and presented with clinical features known to hinder healing. In this context, even a partial reduction in wound size was considered meaningful, as complete closure is often not achievable. The ≥ 50% cutoff was consistent with clinical expectations and expert consensus for this patient subgroup.

Subjects' discomfort and mobility progressively improved, as well as vascularisation criteria, which show the angiogenesis promotion by IDRT, a key point in these difficult‐to‐heal wounds.

Only three patients had a wound recurrence at 18 months, indicating long‐term efficacy of IDRT.

The observed complete healing rate at 18 months was 40%, which may appear modest. However, it should be interpreted in light of the complex clinical profile of the patients included in this study.

Our study population consisted of patients who had failed or were not candidates for early venous interventions as described in the EVRA and ESCHAR trials, thus representing a more refractory cohort.

All patients presented with chronic leg ulcers unresponsive to conventional treatments, and most were considered unsuitable for compression therapy due to local conditions (e.g., exposed tendons or insufficient granulation) or systemic comorbidities (e.g., severe arterial insufficiency, cardiac failure). Compression therapy, the standard of care for purely venous ulcers, was not applied systematically because the population was heterogeneous and included mixed aetiology ulcers. Furthermore, the study aimed to evaluate dermal regeneration in difficult‐to‐treat wounds, not standard venous ulcers.

In this high‐risk population, partial wound healing and reduced ulcer size represent clinically meaningful improvements. Our findings are consistent with prior reports on complex ulcers treated with dermal matrices, which often show variable but limited rates of complete closure. Nonetheless, the benefits of IDRT may extend beyond closure rate, including improved wound bed quality, reduced pain and better long‐term tissue integration.

The evaluation of the QoL and patient's well‐being showed an increase over time. These results are consistent with the surgeon and patient satisfaction. Few device‐related AEs, all of which were localised infections, were reported.

CLUs are frequently a combination of trauma associated with medical comorbidities [21, 22]. Wound healing has been arbitrarily divided into three phases: inflammation, proliferation and maturation, with some authors adding haemostasis as the inciting phase [1]. Healing process abnormalities could be associated with the chronicity, such as decreased cellular migration and proliferation, altered extracellular matrix [23], cytokines and growth factors [24] as well as a deficit in myofibroblast differentiation. Chronic wounds often report an altered number and distribution of macrophages and T cells. Also, macrophages seem abnormal or dysfunctional [25]. This leads to a prolonged inflammatory response and elevated protease activity that damages the extracellular matrix, which cannot support wound healing [26]. IDRT seems to be efficient due to its ability to replace absent or dysfunctional extracellular matrix [27] and to reduce protease activity [28].

The wound closure is crucial in CLUs because the longer the time of wound healing, the greater the risk of developing local and systemic complications. Furthermore, patients with leg ulcers often report a range of physical and psychological symptoms [29, 30, 31], such as pain, fatigue and depression. Thu Do et al. [32] demonstrated the far‐reaching influence of these symptoms on physical and mental health QoL and our results confirm that the treatment of CLUs with IDRT promotes wound closure and consequently the QoL of patients.

TcPO_2_ and laser Doppler measurement demonstrated no significant changes probably due to the origin of the ulcers, mostly venous.

Effective treatments for chronic ulcers could substantially improve patient's QoL and reduce the economic impact of CLUs on health services [33]. Indeed, health care providers and surgeons should consider not only the high costs of bioengineered products but also the total care costs when deciding about the appropriate treatment of a chronic ulcer [34].

As Ryan et al. reported, IDRT used in severely burned adults was associated with a marked decrease in patients' length of stay and hence in health care costs [35], due to its ability to speed up the healing process. Analogous results may be extrapolated in CLU treatment with IDRT, even if further studies should be carried out.

Because of the complexity of chronic wounds and the potential impact of concomitant comorbidities, there is no single intervention that can be established as superior for all patients. Several comparative effectiveness studies have been reported, focusing on numerous therapeutic alternatives in CLU treatment, such as advanced wound dressings [36], vacuum‐assisted closure therapy [37], bioengineered skin grafts [38] and dermal templates [39, 40].

Previous studies have established that IDRT promotes wound closure, resulting in a more consistent and rapid healing of lower extremity ulcers [41]. Dermal templates could be an effective alternative to flap or full‐thickness skin graft for covering deep wounds in limbs to reduce surgical morbidity, especially in debilitated patients with no clinical conditions to undergo a long‐duration procedure [42].

Skin‐grafting is widespread in chronic ulcers treatment. Nevertheless, full‐thickness skin grafts presuppose the creation of a deep wound in the donor site, susceptible to complications such as infections and scar retractions. Additionally, it precludes the use of the same donor site for further grafts. For this reason, partial‐thickness skin grafts are preferred, but they tend to shrink and lead to unsatisfying results [43]. That is mostly due to the paucity or absence of dermis in the partial‐thickness skin graft [44, 45]. Therefore, the use of dermal substitutes associated with thin skin grafts could be considered as the ‘gold standard’ in the treatment of complex leg ulcers [43, 45].

In addition, IDRT seems particularly suitable in those wounds with a compromised vascularisation and low tissue oxygenation. Indeed, as Michot et al. [46] demonstrated, chronic ischemia does not appear to hinder healing with IDRT, since the matrix seems to be less demanding in terms of vascular wound bed and degree of oxygenation than a conventional skin graft.

Several studies have highlighted the utility of IDRT in achieving a faster and better healing of chronic wounds [47], but there are few reports focusing on the use of IDRT for the treatment of chronic leg ulcers, especially prospective studies with a long‐term follow‐up.

The main strength of this study is that it is a prospective study with a relatively long‐term follow‐up, while most of the studies concerning IDRT application in CLUs are either retrospective or focused on ulcers [48]. Moreover, the wound area assessment was carried out through a computerised system that allowed for greater accuracy. Nevertheless, some limitations of this study must be pointed out, such as the small sample size, the non‐comparative nature of the study and the lack of a cost analysis.

These findings contribute valuable insights into the real‐world performance of the intervention and may support more cost‐effective decision‐making in chronic wound care. Furthermore, recently an article by Portincasa et al. [49] brought to light a differentiation between a ‘dermal substitute’ and a ‘bioinductor’. Specifically, the authors underline how only IDRT can be considered a dermal substitute that leads to the formation of neodermis similar to native dermis, while the other scaffolds on the market, composed only of derivatized hyaluronic acid or only collagen of porcine or bovine origin or scaffolds of synthetic origin, must be considered ‘bioinductors’ of granulation tissue since they act only temporarily for a too short a time and are degraded too rapidly by enzymatic activity, bringing about wound contraction. The ability of IDRT to create neodermis, compared to the other collagen‐composed scaffolds, is related to the presence of a glycosaminoglycan, chondroitin‐6‐sulphate. It is a component of ECM, causes the structural integrity of the tissue, allows interactions between cells and ECM, increases the resistance of the scaffold against the action of collagenases and has anti‐inflammatory properties.

## 5 Conclusion

This study demonstrates that the combination of IDRT and split‐thickness skin grafting is a feasible and effective therapeutic approach for the management of chronic leg ulcers, especially in patients with ulcers refractory to conventional treatments. IDRT conforms to the wound bed, encourages wound closure by speeding up the healing process, is easy to use and can even be placed over bone or tendons. The use of IDRT led to successful results in a large majority of patients with CLU, without the complications associated with full thickness grafts donor site or flap procedures.

## Disclosure

The dermal regeneration templates used were provided as part of routine hospital procurement; no commercial entity contributed materials or financial support.

## Ethics Statement

Approved by each participating institution's Institutional Review Board. Protocol RECON‐EMEA‐10 (NCT01285973).

## Conflicts of Interest

The authors declare no conflicts of interest.

## Data Availability

The data that support the findings of this study are available from the corresponding author upon reasonable request. Due to the nature of the data (clinical and patient‐sensitive), restrictions apply to their public availability.

## Endnotes

^1^
All ulcers were classified as venous in aetiology based on duplex ultrasonography. The mention of diabetes or microangiopathy in the figure captions refers to associated comorbidities, not to the primary ulcer aetiology.
